# Promoting physical therapists’ of research evidence to inform clinical practice: part 1 - theoretical foundation, evidence, and description of the PEAK program

**DOI:** 10.1186/1472-6920-14-125

**Published:** 2014-06-25

**Authors:** Julie K Tilson, Sharon Mickan

**Affiliations:** 1 Division of Biokinesiology and Physical Therapy, University of Southern California, 1540 Alcazar St., CHP 155, Los Angeles, CA 90033, USA; 2 Nuffield Department of Primary Care Health Sciences, University of Oxford, Radcliffe Observatory Quarter, Oxford OX2 6GG, UK

**Keywords:** Evidence based practice, Knowledge translation, Education, Post-graduate training, Physical therapy

## Abstract

**Background:**

There is a need for theoretically grounded and evidence-based interventions that enhance the use of research evidence in physical therapist practice. This paper and its companion paper introduce the Physical therapist-driven Education for Actionable Knowledge translation (PEAK) program, an educational program designed to promote physical therapists’ integration of research evidence into clinical decision-making. The pedagogical foundations for the PEAK educational program include Albert Bandura’s social cognitive theory and Malcolm Knowles’s adult learning theory. Additionally, two complementary frameworks of knowledge translation, the Promoting Action on Research Implementation in Health Services (PARiHS) and Knowledge to Action (KTA) Cycle, were used to inform the organizational elements of the program. Finally, the program design was influenced by evidence from previous attempts to facilitate the use of research in practice at the individual and organizational levels.

**Discussion:**

The 6-month PEAK program consisted of four consecutive and interdependent components. First, leadership support was secured and electronic resources were acquired and distributed to participants. Next, a two-day training workshop consisting of didactic and small group activities was conducted that addressed the five steps of evidence based practice. For five months following the workshop, participants worked in small groups to review and synthesize literature around a group-selected area of common clinical interest. Each group contributed to the generation of a “Best Practices List” - a list of locally generated, evidence-based, actionable behaviors relevant to the groups’ clinical practice. Ultimately, participants agreed to implement the Best Practices List in their clinical practice.

**Summary:**

This, first of two companion papers, describes the underlying pedagogical theories, knowledge translation frameworks, and research evidence used to derive the PEAK program – an educational program designed to promote the use of research evidence to inform physical therapist practice. The four components of the program are described in detail. The companion paper reports the results of a mixed methods feasibility analysis of this complex educational intervention.

## Introduction

Physical therapists report positive attitudes about the use of research evidence to inform clinical decision-making [1-3]. Further, most consider evidence-based practice (EBP) important for continuing development of the profession [1] and for quality patient care [4]. Yet, there is a gap between research and practice [5]. Thomas *et al.*[6] found that physical therapists had difficulty integrating research evidence into care for persons with acute hip fracture; instead therapists relied almost exclusively on colleagues and previous experience for clinical decision-making. Likewise, Fritz *et al.*[7] found that only 40% of care for individuals with low back pain was guideline-adherent. While the gap might be closed, in part, by educational efforts, many educational programs designed to enhance the use of research evidence in clinical decision-making have had limited impact on clinical practice [3,8,9]. Hence, there is a gap, not only between research and practice, but between physical therapists’ desire to use research in clinical decision-making and the reality of doing so.

There is a need for theoretically grounded and evidence-based interventions that enhance the use of research evidence in physical therapist practice. This paper and its companion paper [10], introduce the Physical therapist-driven Education for Actionable Knowledge translation (PEAK) program, an educational program designed to promote physical therapists’ integration of research evidence into clinical decision-making. This paper describes the pedagogical theory, knowledge translation (KT) frameworks, and research evidence used to design the program. These foundational underpinnings are followed by a detailed description of the learning objectives and educational methods used. The companion paper [10] reports the results of a mixed methods feasibility assessment of the PEAK program.

## Background

Identification of the theoretical foundations for the PEAK program will enhance our ability to understand and interpret program outcomes. Pedagogical foundations informed how the program promoted learning among individual therapists and KT frameworks informed how the program promoted organizational change. Further, the program design was influenced by careful review of evidence from previous attempts to impact individual and organizational implementation of research evidence. In the paragraphs that follow we introduce the pedagogical foundations, KT frameworks, and research evidence used to derive the PEAK program.

### Pedagogical foundations

The pedagogical foundations for the PEAK program include Albert Bandura’s social cognitive theory and Malcolm Knowles’s adult learning theory. Social cognitive theory posits that observational learning, social experience, and an inner reflective ability are important for the development of self-efficacy. Self-efficacy is the belief in one’s capabilities to organise and act to succeed in particular situations; which in turn influence the way goals, tasks, and challenges are approached [11]. Further, perceived self-efficacy is thought to influence individual’s choice of tasks, the amount of effort invested, their persistence, and their level of confidence [12]. The PEAK program provides opportunities for learners to observe and interact with experts and each other as they learn and explore new skills. Guided small group learning is used to promote participants’ self-efficacy for sustained use of research evidence in practice.

The PEAK program was also influenced by adult learning theory which emphasises problem-based and collaborative learning. Malcolm Knowles made explicit assumptions about adult learning which have formed the basis of graduate and postgraduate education [13]. In essence, adults are most often independent and self-directed learners who draw upon their own experiences to aid their learning. They are motivated to learn by internal drives, and when learning is immediate, relevant, and practical. The PEAK program provided opportunities for self-directed, independent learning where learners selected a topic that had immediate relevance to their clinical practice. Learners then identified and critically appraised research evidence to inform their on-going clinical practice.

### Theoretical frameworks for KT

The process of using research evidence to inform clinical practice is recognized as complex and requiring an understanding of both the knowledge itself and the accompanying interpersonal and social interactions associated with KT [14]. Two complementary frameworks were used to inform the PEAK program’s approach to facilitating KT. The first framework, Promoting Action on Research Implementation in Health Services (PARiHS), posits that successful research utilisation is a function of three elements: the qualities of the context in which the evidence is being used; the nature and type of the evidence; and the facilitation methods used [15,16]. The PEAK program emphasized a culture of management and resource support for research implementation; educated and facilitated participants to use the best available research evidence to address a clinical problem; and required participants to identify observable, evidence-based behaviours that could be incorporated directly into practice.

The second framework, Knowledge to Action (KTA) Cycle, describes a cycle of steps for translating knowledge into clinical action (knowledge creation, problem identification, local adaptation, assessment of barriers, implementation, monitoring, and sustained use) that is underpinned by planned action theory and stakeholder involvement [17]. The PEAK program engaged learners in each step from knowledge creation to implementation*.* Participants created a locally adapted list of actionable behaviors by applying research evidence within their local context. Barriers were addressed through a range of online resources, expert guidance, and support from clinician managers. Ultimately, all participants agreed to implement a defined list of actionable, evidence-based behaviors. The PARIHS and KTA frameworks have been used in a similar complementary manner in previous studies that facilitate knowledge use in clinical practice [15].

### Research evidence for teaching KT and EBP

Finally, the PEAK program was informed by research evidence from previous studies of KT and EBP education, with a focus on those involving physical therapists. Elements from successful programs were borrowed [8,18-21], care was taken to avoid replication of less successful programs [3,8,18,19,22-24], and effort was made to address recognised barriers for physical therapists’ implementation of research evidence [2,4,25].

A systematic review of KT interventions for rehabilitation professionals found that the use of multi-component interventions (e.g. educational workshops, outreach visits, small group work) resulted in improved self-perceived knowledge, and positive practice behaviour change compared to passive dissemination strategies [8]. A recent Cochrane review [26], showed that the effectiveness of educational meetings for healthcare professionals could be enhanced by using mixed interactive and didactic teaching formats. Similarly, in a systematic review of postgraduate evidence-based medicine teaching, integration with clinical practice and use of clinical problems was effective for improving knowledge and patient care [18]. Further, two individual studies with multifaceted interventions including a didactic component, provision of online resources, and small group work showed improvement among medical students in EBP self-efficacy [20] and strength and quality of evidence used in clinical practice [27]. Based on this evidence, the PEAK program was multi-faceted (ie. workshop, electronic resources, guided learning, small group work) and had learners address a group-selected problem relevant to their clinical practice.

Specific attention was also given to addressing established barriers to research utilization in physical therapy. Barriers can be divided into those associated with the organization, the therapists themselves, and the research evidence [2]. The PEAK program was designed to address the following barriers: lack of time (both inefficiency and low prioritization of time required for EBP) [2,4,28]; therapist skills (finding, appraising, interpreting, and applying research evidence) [2,4]; organizational resources (access to computers, the internet, and journal articles) [2]; and organizational culture (lack of organizational support and peer communication) [2]. The PEAK programme was designed to emphasize efficiency and to demonstrate the value of time spent on KT by focusing on clinically important problems that can be informed by research evidence. The program was also designed to improve therapists’ skills, to organize and provide easy access to online and expert resources, and to promote a culture of managerial and peer to peer support for using research in practice.

By founding the PEAK program on pedagogical theory, established KT frameworks, and a growing body of research evidence, we are better prepared to scrutinize, understand, and interpret results from the initial feasibility study of the program reported in the companion paper [10].

## Discussion

The overall goal of the PEAK program was to promote physical therapists’ integration of research evidence into clinical decision-making at the individual (i.e., EBP) and organizational (i.e., KT) level. The 6-month program was designed to enhance attitudes, knowledge, skills, and EBP behaviors among individual therapist participants and to promote KT between geographically separate facilities in a single healthcare organization.

### PEAK learning objectives

The learning objectives for individual participants cover all five steps defined for EBP (ask, search, appraise, integrate, evaluate) [29]. By the end of the intervention we expected that therapists would be able to:

1. Identify gaps in knowledge and develop focused, searchable clinical questions;

2. Find the best available evidence to inform their question using appropriate online databases;

3. Critically appraise the quality of found evidence;

4. Write succinct statements of locally recommended practices that integrate research evidence with their clinical expertise and knowledge of patient perspective; and

5. Integrate newly learned skills and behaviors into their everyday work habits.

Additionally, from an organizational perspective, we expected that, at the conclusion of the PEAK program, all therapists would:

1. Agree to follow the common set of locally generated, evidence-based, best practices that they had developed, for a specific group-selected patient population;

2. Be prepared to engage in future activities that facilitate the use of research to inform clinical practice; and

3. Demonstrate implementation of research within their clinical practice.

### Educational components

**Figure 1:**
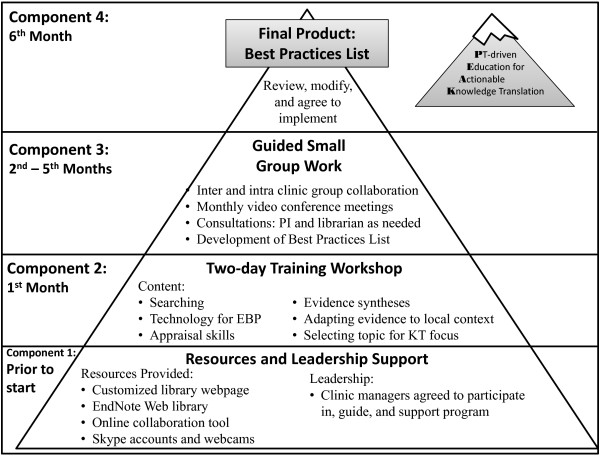
**Timing and integration of components of the Physical Therapist-driven Education for Actionable Knowledge Translation (PEAK) program (figure reads from bottom to top).** The program started with garnering support from clinic managers and placing links to technology resources at each facility’s computer work stations. Next, participants attended a two-day workshop addressing evidence based practice (EBP) and knowledge translation (KT) skills. Five months of guided small group work followed as participants developed the Best Practices List. In the final month, the Best Practices List was reviewed by unaffiliated expert faculty. Finally, after multiple rounds of revisions, all participants agreed to implement the Best Practices List in their clinical practice.

The PEAK program consisted of four consecutive and interdependent components (Figure 1):

1. Securing resources and leadership support

2. A two-day training workshop

3. Guided small group work to develop a locally relevant list of evidence-based actionable behaviors – the “Best Practices List”

4. Review, modification, and agreement to implement the Best Practices List

#### Component 1

Prior to starting the PEAK program leadership support was secured by engaging managers of the three, geographically separate physical therapy service departments (2 outpatient, 1 inpatient) at the University of Southern California (USC) to contribute to logistical organization of the PEAK program and to participate throughout the program. Resources for supporting the integration of research in practice were provided to all participants as follows:

•Custom library web page – developed and maintained by a medical librarian to reflect key online resources

•Group online reference manager account (EndNote Web® [Thompson Reuters])

•Online collaboration tool (Backpack™, 37 Signals, LLC) – purchased and set-up for all participants to use (a research assistant managed organization of the collaboration tool)

•Skype™ (Microsoft Skype Division) accounts – established for each facility, including purchase and installation of webcams to facilitate inter-facility web conferencing

Links to online resources were installed as bookmarks on each participant’s work computer.

#### Component 2

During the first month of the program participants attended a two-day workshop that combined didactic and active learning around topics of EBP and KT including:

•Review of the 5-step EBP model (1 hour)

•Searching skills (3 hours; PubMed, National Guidelines Clearinghouse, Translating Research Into Practice Database [TRIP], Physiotherapy Evidence Database [PEDro])

•Appraisal skills (3 hours; primary studies of interventions, systematic reviews, and clinical practice guidelines)

•Integrating research evidence with patient perspectives and clinical expertise (1 hour)

•Using technology to keep up to date (2 hours: podcasts, myNCBI auto-searches, RSS feeds, etc.; study-specific tools: Backpack™, EndNote Web®, Skype™)

•Selection of clinical area and five sub-topics around which a list of locally relevant evidence-based best practices would be generated (2 hours)

•Initiation of small group work for developing the Best Practices List (2 hours)

A librarian attended one day of the workshop to promote participants’ use of library resources and was available for consultation throughout the course of the program. A copy of the educational materials used for the 2-day workshop is available from the corresponding author.

It is important to note that the *participants* selected the clinical area that would be pursued for the rest of the program based on their common interests and a perceived opportunity for patient benefit. Further, participants identified five sub-topics of the clinical area and organized themselves into five corresponding small groups based on the sub-topic(s) of greatest interest to each participant.

#### Component 3

For 5 months following the workshop, participants met regularly in small groups (three to seven therapists) to develop a list of locally relevant ‘best practices’ for their clinical sub-topic. A designated group leader accepted responsibility for organizing regular small group communication and monthly reporting to the larger group. Each small group worked through the five EBP steps to find, appraise, and synthesize the highest quality research evidence for their clinical sub-topic. More specifically, groups were tasked to use research evidence, their own expertise, and knowledge of patient perspectives to generate actionable, evidence-based behaviors that could be implemented in their own practice. Actionable, evidence-based behaviors submitted by each small group were compiled into a single, “Best Practices List” for all participants to implement.

Small groups determined how often they met (virtually or in person) and used the online collaboration tool to accomplish their work. Monthly lunchtime meetings were conducted using Skype™ video conference for all participants to report on and discuss their progress. Monthly meetings were facilitated by the study principal investigator (PI) and attended by the study librarian. The study principal investigator and librarian met individually with groups when requested.

#### Component 4

At the end of the 5^th^ month, each small group submitted between 7 and 15, actionable, evidence-based behaviors to the Best Practices List. The study PI compiled the behaviors and distributed them to all participants for review and comment. Two rounds of review and comment were conducted online. Next, the list was sent for external review by experts selected by participants. Expert feedback was incorporated into the Best Practices List and, at the end of the 6^th^ month, participants attended a final two hour meeting to review and discuss each behavior. Edits were made until all participants were satisfied that they could adhere to the recommended practice. At the conclusion of this final meeting the study participants gave verbal affirmation that they agreed with and would follow the behaviors outlined in the Best Practices List. This final list (Additional file 1) was published in booklet form and distributed electronically and in hard copy to all participants.

##### Instructor

The instructor for the program was the study PI (JKT) – a physical therapist with 10 years experience teaching EBP and promoting KT in clinical and classroom environments.

## Summary

Nutley *et. al.* emphasize the need to develop a coherent framework for developing multifaceted approaches to promoting the use of research in practice [14]. This paper has presented a detailed description of the component activities within the PEAK programme, together with a comprehensive theoretical and evidence-based explanation of key mechanisms that underpin these strategies. Table 1 illustrates how components of the PEAK program link to social cognitive theory and adult learning theory and to the PARIHS and KTA frameworks. Table 2 illustrates how components of the PEAK program link to research evidence about educational programs designed to improve the use of research evidence in practice.

**Table 1:** Pedagogical theories and knowledge translation frameworks used to inform the PEAK program

**Concept from theory or framework**	**Element of PEAK program**
***Pedagogical foundations***	
Observational learning, social experience, and an inner reflective ability are important in the development of self-efficacy [11].	All aspects of PEAK were designed to foster self-efficacy through facilitated step-by-step procedures that offered multiple opportunities for learning. Participants had the opportunity to learn with and from each other in small groups and using the online collaboration tool.
Self-efficacy is reinforced through personal performance, verbal persuasion from credible sources, and observations of others [12].	Individual success in searching for and critically appraising research evidence was shared in small groups and affirmed by the program experts and peers in monthly conferences.
Adults are independent and self-directed learners who draw upon their own experiences to aid their learning [13].	Groups were given an independent task—to develop a Best Practices List around a group-selected clinical problem. They accessed resources as required from a range of online and instructor resources.
Adult learners are motivated to learn by internal drives, when learning is immediate, relevant, and practical [13].	The process of developing the Best Practices List focused on a clinical problem selected by participants as relevant to their collective practice.
***Knowledge translation frameworks***	
PARiHS: Successful implementation is a function of the qualities of the context in which the evidence is being used [15,16].	Leadership support was secured by encouraging all managers to participate in logistical organization and in the educational program. A physical barrier of three geographic locations was acknowledged and addressed using online resources.
PARiHS: Successful implementation is a function of the facilitation methods used [15,16].	Regular communication in small groups was driven by the need for monthly reporting. Additional support from the study librarian and PI was available to all participants on request.
KTA: Key steps include:knowledge creation, problem identification, local adaptation, assessment of barriers, implementation, monitoring, and sustained use [17].	Participants adapted research knowledge to their local environment, using an awareness of key barriers for a group-selected clinical problem. They then agreed that sustained use would be monitored via audits of medical record reporting.

*Abbreviations*: PEAK – Physical therapist-driven Education for Actionable Knowledge translation; PARiHS – Promoting Action on Research Implementation in Health Service; KTA – Knowledge to Action; PI – Principal Investigator.

**Table 2:** Research evidence used to inform the PEAK Program

**Research evidence**	**Element of PEAK program**
***Characteristics of effective educational programs***	
Combined interactive and didactic components [26]	Participants attended a 2-day didactic workshop and monthly 1-hour educational sessions in addition to working in small groups.
More intense and with more serious implications [26]	The 6-month nature of this program was designed to be intensive. Having participants select a topic for the Best Practices List ensured that it was perceived as important.
Clinically integrated [18]	Use of participant-driven areas of clinical interest was designed to promote a direct link between the program and patient care.
Participant-driven and multi-faceted [8]	The range of online resources, formal workshop, and support for learning in small groups—all around participant-selected topics—provided a multi-faceted, participant-driven learning environment.
***Known Barriers to EBP***	
Efficiency [28]	The PEAK program is designed to increase efficiency by building individual participant skills and by optimizing organizational resources.
Individual therapist skills [2,4]	The 2-day workshop taught and reinforced a common, basic set of EBP skills, which individuals needed to contribute to the Best Practices List.
Organizational barriers: Resources [2]	University and library resources were bookmarked on computers in the clinical practice environment.
Organizational barriers: Culture [2]	The initial 2-day workshop required attendance from therapists across all clinical sites. The use of small groups, with individuals from different sites, was designed to facilitate a culture of cooperation around using research evidence. Additionally, clinical managers were actively involved in participating in and supporting the PEAK program.

*Abbreviations*: *PEAK* – Physical therapist-driven Education for Actionable Knowledge translation; EBP – Evidence Based Practice.

Upon developing a new educational program it is important to describe a coherent theoretical framework that explains why and how the program is expected to work. The framework can be then used in an analysis of change—to suggest explanations for why some components worked well together and to identify those components considered to be most important for achieving the program’s learning objectives. Two pedagogical theories have been identified to explain change associated with the PEAK program. The PEAK program highlights the importance of small group achievement of specific tasks (social cognitive theory) and of independent and experiential learning situated in clinical practice (adult learning theory).

Further, two complementary and theoretically compatible KT frameworks were chosen to integrate contemporary understanding of research utilization at the organizational level into the PEAK program. Both frameworks emphasize that local culture and structures will impact the way in which knowledge is implemented. The KTA framework emphasises the importance of both securing leadership and resource support and adapting recommendations with consideration for the local context. The PARiHS framework suggests that successful implementation will only occur when the evidence is robust, when practitioners can access the evidence and agree with it, and when the intervention occurs within a context that is receptive. Additionally, careful planning of the facilitation process is required for participants to integrate the evidence in their local context [30]. Finally, this theoretical framework was applied with reference to the evidence of effective educational interventions and recognised barriers to integrating research into practice for physical therapists and health care professionals in general.

The multifaceted and participant-driven nature of the PEAK program makes it an inherently complex intervention [31]. Recent advice about designing and evaluating complex interventions, suggests that it is important to evaluate the feasibility of both the design and the implementation of a complex intervention before testing it more rigorously in a randomized controlled trial [32]. This process can provide important information about the need to refine the design before embarking on a full scale evaluation [31]. The companion paper to this manuscript reports the results of a mixed methods feasibility analysis of the PEAK program [10].

## Conclusion

This first of two companion papers describes the underlying pedagogical theories, KT frameworks, and research evidence used to derive the PEAK program – an educational program designed to promote the use of research evidence to inform physical therapist practice. The four components of the program are described in detail to support future evaluation and replication of the program. A companion paper reports the result of a mixed methods feasibility analysis of this program.

## Competing interests

The authors declare that they have no competing interests.

## Authors’ contributions

JKT and SM contributed equally to the writing of this manuscript. Both authors read and approved the final manuscript.

## Pre-publication history

The pre-publication history for this paper can be accessed here:

http://www.biomedcentral.com/1472-6920/14/125/prepub

## Supplementary Material

Additional file 1 Best Practices List generated by PEAK participants: “University of Southern California Best Practices List: Physical Therapy for Lumbar Spine Conditions”. Click here for file

## References

[B1] Barnard S Wiles R Evidence-based physiotherapy: physiotherapists’ attitudes and experiences in the Wessex area Physiotherapy 2001 87 115 124

[B2] Salbach NM Jaglal SB Korner-Bitensky N Rappolt S Davis D Practitioner and organizational barriers to evidence-based practice of physical therapists for people with stroke Phys Ther 2007 87 1284 1303 discussion 1304-1286 17684088 10.2522/ptj.20070040

[B3] Schreiber J Stern P Marchetti G Provident I Strategies to promote evidence-based practice in pediatric physical therapy: a formative evaluation pilot project Phys Ther 2009 89 918 933 19643835 10.2522/ptj.20080260

[B4] Jette DU Bacon K Batty C Carlson M Ferland A Hemingway RD Hill JC Ogilvie L Volk D Evidence-based practice: beliefs, attitudes, knowledge, and behaviors of physical therapists Phys Ther 2003 83 786 805 12940766

[B5] Goldstein LB Bushnell CD Adams RJ Appel LJ Braun LT Chaturvedi S Creager MA Culebras A Eckel RH Hart RG Hinchey JA Howard VJ Jauch EC Levine SR Meschia JF Moore WS Nixon JV Pearson TA Guidelines for the primary prevention of stroke: a guideline for healthcare professionals from the American Heart Association/American Stroke Association Stroke 2011 42 517 584 21127304 10.1161/STR.0b013e3181fcb238

[B6] Thomas S Mackintosh S Halbert J Determining current physical therapist management of hip fracture in an acute care hospital and physical therapists’ rationale for this management Phys Ther 2011 91 1490 1502 21817011 10.2522/ptj.20100310

[B7] Fritz JM Cleland JA Brennan GP Does adherence to the guideline recommendation for active treatments improve the quality of care for patients with acute low back pain delivered by physical therapists? Med Care 2007 45 973 980 17890995 10.1097/MLR.0b013e318070c6cd

[B8] Menon A Korner-Bitensky N Kastner M McKibbon KA Straus S Strategies for rehabilitation professionals to move evidence-based knowledge into practice: a systematic review J Rehabil Med 2009 41 1024 1032 19893996 10.2340/16501977-0451

[B9] Bekkering GE van Tulder MW Hendriks EJM Koopmanschap MA Knol DL Bouter LM Oostendorp RAB Implementation of clinical guidelines on physical therapy for patients with low back pain: Randomized trial comparing patient outcomes after a standard and active implementation strategy Phys Ther 2005 85 544 555 15921475

[B10] Tilson J Mickan S Sum J Zibell M Dylla J Howard R Promoting physical therapists’ use of research evidence to inform clinical practice: part 2 - a mixed methods evaluation of the PEAK program BMC Med Educ 2014 14 126 24965574 10.1186/1472-6920-14-126 PMC4080990

[B11] Bandura A Self-efficacy - toward a unifying theory of behavioral change Psychol Rev 1977 84 191 215 847061 10.1037//0033-295x.84.2.191

[B12] Kaufman DM Applying educational theory in practice BMJ 2003 326 213 216 12543841 10.1136/bmj.326.7382.213 PMC1125068

[B13] Knowles MS Andragogy in Action 1984 1 San Francisco: Jossey-Bass

[B14] Nutley S Walter I Davies HTO Promoting evidence-based practice: models and mechanisms from cross-sector review Res Soc Work Pract 2009 19 552 559

[B15] Rycroft-Malone J Harvey G Seers K Kitson A McCormack B Titchen A An exploration of the factors that influence the implementation of evidence into practice J Clin Nurs 2004 13 913 924 15533097 10.1111/j.1365-2702.2004.01007.x

[B16] Kitson AL Rycroft-Malone J Harvey G McCormack B Seers K Titchen A Evaluating the successful implementation of evidence into practice using the PARiHS framework: theoretical and practical challenges Implement Sci 2008 3 1 18179688 10.1186/1748-5908-3-1 PMC2235887

[B17] Graham ID Logan J Harrison MB Straus SE Tetroe J Caswell W Robinson N Lost in knowledge translation: time for a map? J Contin Educ Health Prof 2006 26 13 24 16557505 10.1002/chp.47

[B18] Coomarasamy A Khan KS What is the evidence that postgraduate teaching in evidence based medicine changes anything? A systematic review BMJ 2004 329 1017 1019 15514348 10.1136/bmj.329.7473.1017 PMC524555

[B19] Flores-Mateo G Argimon JM Evidence based practice in postgraduate healthcare education: A systematic review BMC Health Serv Res 2007 7 8 17655743 10.1186/1472-6963-7-119 PMC1995214

[B20] Allan GM Korownyk C Tan A Hindle H Kung L Manca D Developing an integrated evidence-based medicine curriculum for family medicine residency at the University of Alberta Acad Med 2008 83 581 587 18520465 10.1097/ACM.0b013e3181723a5c

[B21] Straus SE Ball C Balcombe N Sheldon J McAlister FA Teaching evidence-based medicine skills can change practice in a community hospital J Gen Intern Med 2005 20 340 343 15857491 10.1111/j.1525-1497.2005.04045.x PMC1490095

[B22] McCluskey A Lovarini M Providing education on evidence-based practice improved knowledge but did not change behaviour: a before and after study BMC Med Educ 2005 5 40 16364181 10.1186/1472-6920-5-40 PMC1352357

[B23] Stevenson K Lewis M Hay E Do physiotherapists’ attitudes towards evidence-based practice change as a result of an evidence-based educational programme? J Eval Clin Pract 2004 10 207 217 15189387 10.1111/j.1365-2753.2003.00479.x

[B24] Taylor RS Reeves BC Ewings PE Taylor RJ Critical appraisal skills training for health care professionals: a randomized controlled trial [ISRCTN46272378] BMC Med Educ 2004 4 30 15585061 10.1186/1472-6920-4-30 PMC539272

[B25] Bridges PH Bierema LL Valentine T The propensity to adopt evidence-based practice among physical therapists BMC Health Serv Res 2007 7 103 17615076 10.1186/1472-6963-7-103 PMC1929067

[B26] Forsetlund L Bjorndal A The potential for research-based information in public health: Identifying unrecognised information needs BMC Public Health 2001 1 1 11208260 10.1186/1471-2458-1-1 PMC29105

[B27] Straus S, Richardson S, Glasziou P, Haynes B Evidence-Based Medicine: How to Practice and Teach EBM 2005 Philadelphia: Churchill Livingston

[B28] Bayley MT Hurdowar A Richards CL Korner-Bitensky N Wood-Dauphinee S Eng JJ McKay-Lyons M Harrison E Teasell R Harrison M Graham ID Barriers to implementation of stroke rehabilitation evidence: findings from a multi-site pilot project Disabil Rehabil 2012 34 1633 1638 22631218 10.3109/09638288.2012.656790

[B29] Dawes M Summerskill W Glasziou P Cartabellotta A Martin J Hopayian K Porzsolt F Burls A Osborne J Sicily statement on evidence-based practice BMC Med Educ 2005 5 1 15634359 10.1186/1472-6920-5-1 PMC544887

[B30] Rycroft-Malone J Seers K Chandler J Hawkes CA Crichton N Allen C Bullock I Strunin L The role of evidence, context, and facilitation in an implementation trial: implications for the development of the PARIHS framework Implement Sci 2013 8 28 23497438 10.1186/1748-5908-8-28 PMC3636004

[B31] Craig P Dieppe P Macintyre S Nazareth I Petticrew M Developing and evaluating complex interventions: the new Medical Research Council guidance BMJ 2008 337 a1655 18824488 10.1136/bmj.a1655 PMC2769032

[B32] Campbell NC Murray E Darbyshire J Jon E Farmer A Griffiths F Guthrie B Lester H Wilson P Kinmonth AL Designing and evaluating complex interventions to improve health care BMJ 2007 334 455 459 17332585 10.1136/bmj.39108.379965.BE PMC1808182

