# Supplementary material for: Promoting physical therapists’ of research evidence to inform clinical practice: part 1 - theoretical foundation, evidence, and description of the PEAK program
Source: BMC Med Educ. 2014 Jun 25;14:125. doi: 10.1186/1472-6920-14-125 (PMC4085660; doi:10.1186/1472-6920-14-125)
Supplement: Additional file 1 — Best Practices List generated by PEAK participants: “University of Southern California Best Practices List: Physical Therapy for Lumbar Spine Conditions”. [file 1472-6920-14-125-S1.pdf]

University of Southern California
Best Practices List:
Physical Therapy for Lumbar Spine Conditions

Mission: To enhance and standardize evidence-based practice for patients with spine conditions receiving physical therapy at the University of Southern California

Date Developed: 1/2011 through 6/2011

Contributors: Andrea Austin, PT, DPT, OCS, ATC, CSCS; Geoff Cariker, DPT; Robert Cook, MPT; Jackie Dylla, DPT; Janelle Gilmer, PT, DPT; Lori Ginoza, PT, DPT, NCS; Susan Hartling, PTA; Erin Hayden, PT, DPT, OCS; Julie Hershberg, DPT, NCS; Robbin Howard, PT, DPT, NCS; Sean Johnson, PT DPT OCS; Yas Kasayama, PT, DPT, MHA, OCS; Yogi Matharu, DPT, OCS; Amy Pomrantz, PT, DPT, OCS, ATC; Elise Ruckert, PT, DPT, NCS; Scott Russell, PT, DPT, GCS; Raymond Streeter, PTA; Jonathan Sum, DPT, OCS, SCS, CSCS; Julie Tilson, DPT, MS, NCS; Kimiko Yamada, PT, DPT, OCS, ATC, CSCS; Qin Yao, PT; Maria Zibell, PT, MPT

Investigators: Julie Tilson, DPT, MS, NCS; Sharon Mickan, PhD, OT; Jonathan Sum, DPT, OCS, SCS, CSCS; Maria Zibell, PT, MPT; Jackie Dylla, DPT; Robbin Howard, PT, DPT, NCS

Expert Reviewers: Joe Godges, DPT ; Daniel Kirages, PT, DPT, OCS, FAAOMPT; Kornelia Kulig, PhD, PT, FAPTA; Mike O'Donnell, PT, DPT, OCS, FAAOMPT; Marisa Perdomo, PT, DPT

Research Assistant: Adrianne Greene, SPT

Table of Contents

Outcome Measures……………………………………………………………………….3

Stenosis…………………………………………………………………………………..4

Spine Tumors……………………………………………………………………………7

Non-specific Low Back Pain…………………………………………………….10

Post-Surgical Lumbar Spine…………………………………………………….13

Outcome Measures

Parentheses indicate the overall strength of the recommendation.

Behaviors	
1	For patients with LBP including lumbar stenosis the modified Oswestry Disability Index should be administered at the beginning and end of treatment.* Goals should specify a minimum change of 6 points and review of progress should identify whether this minimum important change was achieved. [1-4] (Strong)
2	For patients with LBP the Fear Avoidance Beliefs Questionnaire should be administered at the beginning and end of treatment. [4] *(Strong)
3	All patients with LBP should be asked the following two questions (1) "During the past month, have you often been bothered by feeling down, depressed, or hopeless?" and (2) "During the past month, have you often been bothered by little interest or pleasure in doing things?" to screen for depression. Results should be documented and acted upon based on the therapists' discretion. [5] (Strong)

*Beginning and end of treatment presumes a course of physical therapy care exceeding 1-2 sessions. For very brief episodes of care, a single assessment may be appropriate.

1. Ostelo RW, Deyo RA, Stratford P, et al. Interpreting change scores for pain and functional status in low back pain: towards international consensus regarding minimal important change. *Spine (Phila Pa 1976).* Vol 33. United States2008:90-94.

2. Fritz JM, Irrgang JJ. A comparison of a modified Oswestry Low Back Pain Disability Questionnaire and the Quebec Back Pain Disability Scale. *Phys Ther.* Feb 2001;81(2):776-788.

3. *Diagnosis and treatment of degenerative lumbar spinal stenosis.* Burr Ridge, IL: North American Spine Society;2007.

4. Delitto A, George SZ, van Dillen L, Sowa G, Godges JJ. Low back pain clincial practice guidelines linked to the International Classification of Functioning, Disability, and Health. *J Orthop Sports Phys Ther.* in process.

5. Whooley MA, Avins AL, Miranda J, Browner WS. Case-finding instruments for depression. Two questions are as good as many. *J Gen Intern Med.* Jul 1997;12(7):439-445.

Stenosis

Parentheses indicate the overall strength of the recommendation.

Behaviors	
1	For patients with lumbar stenosis, a limited course (2x/week for 6 weeks, 8-12 visits) of PT that includes exercise should be provided. If this is not provided, the reason should be documented.[1,2]<br><br>***Reviewer Comments:*** *The 6 week course allows for potential physiological healing of tissues just with time alone. Through therapeutic interventions we desire to create tissue changes that may lead to post-treatment soreness. A frequency of 2x/week is appropriate to allow enough time to recover from the typical 24-48 hour soreness and still have time that week to do it again. A realistic prognosis should be made during the initial evaluation and this discussed in a collaborative manner with the patient.*
2	For patients with lumbar stenosis, the initial PT session and subsequent sessions if needed should include education about L/S flexion exercises and lower extremity stretching. Lumbar spine flexion exercises should include: William's flexion exercise of single knee to chest, double knee to chest, and hooklying bilateral LE rotation. Lower extremity stretching should include stretches to hip flexors and hamstrings. . If none or only a small number of these are provided the therapist should document the reason.[1,3]<br><br>***Reviewer Comments:*** *Education is an important component early in the course of treatment. If patients understand what is potentially happening to their body and how making the dedicated effort to attend and participate in physical therapy services will help them, then their compliance and attitude will be positive. This only helps with appropriate central processing to assist with symptom improvement. Home exercise program prescription is the most important component of the intervention plan because it will carry through with the patient beyond the clinical care and establish independent symptom management.*<br><br>*Mechanically the flexion based exercises have been shown to be helpful and a good first line intervention after a thorough examination. Especially since time will be limited for intervention during the initial PT session. The program listed above covers all the important components necessary to achieve symptom relief and symptom prevention.*
3	For patients with lumbar stenosis, the initial PT session should include education about an introduction to a walking program. Initiation of a full weight bearing walking program for HEP will depend on the patient's irritability level. If this is not provided, the reason should be documented.[1]<br><br>***Reviewer Comments:*** *A walking program can be introduced, but irritability data must be collected carefully to know if the patient will tolerate even brief walking. The clinician may want to initiate a full weight bearing walking program after intervention has been performed that allow for tissue changes to occur that will provide the patient with better tolerance for a walking program.*

4	Patients with lumbar stenosis involved in a comprehensive PT program should be offered body-weight supported treadmill training or stationary cycling. Therapists should document that this choice was offered and the decision should reflect consideration of patient preference, clinical expertise, and equipment availability.[4]<br><br>***Reviewer Comments:*** *The literature points to these two activities being comparable and helpful in the lumbar stenosis population. It is important to maintain mobility of the lower extremities, primarily the hips. Gycling allows for forward flexion posture. Body weight supported treadmill reduces compressive forces acting upon the lumbar spine. Therefore, in each activity tolerance to exercise is achieved.*<br><br>*The clinician will have to make the decision of when to incorporate these activities into the treatment plan. One recommendation is after manual therapy has allowed for an increase in passive mobility at both lumbar and hip regions. At this point the body can be more ready to maximize the hip motion (tolerated extension) during the walking, as well as achieve better segmental lumbar flexion during the cycling.*
5	For patients with lumbar stenosis receiving body-weight supported (BWS) treadmill training, the BWS should minimize the patient's symptoms and allow the patient to ambulate as comfortable as possible. Over subsequent sessions, the therapist should reduce the amount of BWS and increase gait velocity and distance as the patient is able. If these progressions are not achieved the therapist should document the limitations preventing progression.[1]<br><br>***Reviewer Comments:*** *This is the typical progression of any intervention plan. The goal is to help the patient achieve functional carry-over into regular life activities without the assistive device of supported walking. The severity of the stenosis will dictate the prognosis of the gait performance progression being realistic. Should the clinician and patient choose to use BWS as an intervention; a realistic prognosis should be made during the initial evaluation and discussed in a collaborative manner with the patient. As with the full-weight bearing walking program, patient's irritability should be highly monitored.*
6	For patients with lumbar stenosis, instruction in abdominal bracing activation and trunk stabilization should be provided for improved pelvic control and lumbar stabilization. If not provided, the therapist should document the reason. [5]<br><br>***Reviewer Comments:*** *Abdominal bracing activation establishes overall pelvic control and lumbar stabilization. This type of abdominal control will allow for disassociation of lower limb movement with lumbopelvic motion which will reduce the opportunity of lumbar extension with simple movements such as walking, transfers, and lifting activities. Perhaps a better suited abdominal exercise to establish overall pelvic control and lumbar stabilization is abdominal bracing activation.*
7	For patients with lumbar stenosis, strengthening exercises should be provided that include gluteal muscle strengthening. If these strengthening exercises are not provided, the therapist should document the reason.[1,3]<br><br>***Reviewer Comments:*** *Gluteal muscle strengthening is imperative for optimal performance of hip extension and return from lumbar flexion movements. Lack of gluteal activation will likely result in over-activity of the lumbar paraspinals which provides an undesired compressive force upon the lumbar spine leading to a potential increase in symptoms. These impairments can be classified as muscle imbalances. Care should be taken to closely monitor gluteal strengthening exercises where the patient can compensate by using lumbar paraspinals.*

8	For patients with lumbar stenosis who require short-term pain relief, the option of using epidural steroid injections as an adjunct to physical therapy should be considered and discussed with the patient and his or her physician. Such consideration should be documented.[6]<br><br>***Reviewer Comments:*** *Conservative management through physical therapy will be the focus our treatment plan. However, in some cases pain issues may be preventing the implementation or tolerance of physical therapy care. This situation may require medical management of epidural steroid injections to reduce the pain and allow tolerance to physical therapy interventions. This multi-disciplinary approach is the hallmark of appropriate patient management throughout the healthcare landscape.*
9	For patients with lumbar stenosis, manual therapy with a flexion bias for the thoracic and lumbar spine should be provided. If manual therapy with a flexion bias is not provided the reason should be documented.[1,3]<br><br>*Examples of these techniques are as follows:*<br>Seated flexion with or without use of a Swiss ball; seated flexion with contralateral rotation; manual movement with mobilization for thoracic and lumbar spine flexion with patient seated with or without Swiss ball; lumbar spine traction with Morgan Harness in supine hooklying or standing; hip joint mobilizations. [7]<br><br>**Reviewer Comments:** The above mentioned manual procedures are appropriate. The purpose of these procedures is to reduce compressive forces upon the lumbar spine. The more movement options allowed by the spine the less opportunity there is for long term compression/extension pressures which will most likely be symptom producing. The intent of these procedures must be determined to decide if there are other procedures to accomplish the same goals. The impairments addressed are mobility deficits (lack of joint play in desirable direction) and flexibility deficits (lack of muscle length to allow desired spine position). Therefore, the clinician can be creative and use other skilled methods desired to address these impairments.

1. Whitman JM, Flynn TW, Childs JD, et al. A comparison between two physical therapy treatment programs for patients with lumbar spinal stenosis - A randomized clinical trial. *Spine.* Oct 2006;31(22):2541-2549.

2. Reiman M, Harris J, Cleland J. Manual therapy interventions for patients with lumbar spinal stenosis: a systematic review. *New Zealand Journal of Physiotherapy.* 2009;37(1):17-28.

3. Whitman JM, Flynn TW, Fritz JM. Nonsurgical management of patients with lumbar spinal stenosis: a literature review and a case series of three patients managed with physical therapy. *Phys Med Rehabil Clin N Am.* Feb 2003;14(1):77-101, vi-vii.

4. Pua YH, Cai CC, Lim KC. Treadmill walking with body weight support is no more effective than cycling when added to an exercise program for lumbar spinal stenosis: a randomised controlled trial. *Aust J Physiother.* 2007;53(2):83-89.

5. Teyhen DS, Rieger JL, Westrick RB, Miller AC, Molloy JM, Childs JD. Changes in deep abdominal muscle thickness during common trunk-strengthening exercises using ultrasound imaging. *J Orthop Sports Phys Ther.* Oct 2008;38(10):596-605.

6. Koc Z, Ozcakir S, Sivrioglu K, Gurbet A, Kucukoglu S. Effectiveness of physical therapy and epidural steroid injections in lumbar spinal stenosis. *Spine (Phila Pa 1976).* Vol 34. United States2009:985-989.

7. Fritz JM, Cleland JA, Childs JD. Subgrouping patients with low back pain: evolution of a classification approach to physical therapy. *J Orthop Sports Phys Ther.* Jun 2007;37(6):290-302.

Spine Tumors

Parentheses indicate the overall strength of the recommendation.

	Behaviors
1	For patients with spine tumors, PT evaluation (chart review and/or subjective history) should include: (1) Prior level of function, (2) Current level of function, (3) Medical diagnosis (location of primary cancer), and (5) Temporal pattern associated with onset of neurologic impairments. (Strong) [1,2]
2	For patients with spine tumors, the initial assessment should include the level and severity (complete vs. incomplete) of injury. It should be clear in the therapist's documentation how this information has informed the treatment plan (e.g., recovery vs. compensation approach). (Strong)[3-5] **1,3,5**
3	In the acute care setting, the patient's oncology team should be consulted regarding prognosis, which should be documented within the initial evaluation or first follow-up note. (Strong) [4,6]
4	For hospitalized patients with spine tumors, heart rate, blood pressure, respiratory rate, pulse oximetry, and pain should be included in the initial assessment. Subsequent routine (each visit for ICU/DOU patients; weekly for Medical/Surgical patients) monitoring of patients with metastatic cord compression should also be documented.[6]<br><br>During intervention, therapists should monitor aggravating factors/easing favors of patient's pain and report significant increases/persistent pain to the MD/primary RN. (Evidence = expert opinion)
5	Rehabilitation goal setting for patients with spinal tumors and poor prognosis should be problem-focused and short term (less than 1 month), to allow patients to maximize time spent at home and with loved ones. These goals should be developed in collaboration with the patient and family. (Strong) [6,7]
6	For patients with post-operative ambulation ability (incomplete injury), PT intervention should focus on maximizing ambulation capacity and functional independence.(Moderate)[2,6,8,9]
7	For patients with spine tumors, a significant increase in pain or change in neurological symptoms during progressive functional mobility program is a red flag for cord or peripheral nerve compression. If such symptoms arise, the patient should be returned to the supine position (or a position where the pain or symptoms are reversed). (Strong) [6]

8	Patients with spine tumors and evidence of postural hypotension should be managed by proper positioning (e.g., reclining chair/bed) and devices (e.g., anti-embolic stockings) to improve venous return. [5,6] Depending on level of completeness/muscle function in lower extremities, lower extremity exercise such as ankle pumps may also improve venous return (Evidence = expert opinion).
9	Patients with spine tumors and injury above T12 should be provided with instruction in respiratory function exercises (including breathing exercises, assisted cough techniques, diaphragmatic strengthening, and positioning) to improve clearance of lung secretions and prevent pulmonary decline. If these exercises are not provided, the reason should be documented. [5,6]
10	Patients with spine tumors, significant residual neurological deficits, functional decline, and a 'good' prognosis (defined by high activity tolerance and strong rehabilitation potential) should be offered referral to a specialized or designated spinal cord injury rehabilitation unit (e.g. SCI Model System Center[*] or CARF Spinal Cord Accreditations[†]). The patient and team's discussion of this option should be documented. If this is not offered the reason should be provided. (Strong)[6,10]
11	For non-ambulatory patients with patients with absent sacral sensation, a history of skin breakdown, and/or with ASIA C, B or A who do not have LE strength adequate for independent standing/scooting, therapists should document at discharge (across the continuum of care): (1) pressure relief education provided, (2) the patient's ability to perform pressure relief (including techniques, positions [bed/wheelchair], adherence, and assistance required), (3) and equipment provided (e.g. pressure relieving cushions, wheelchair recommendations). (Strong)[5-7,11]

1. Fattal C, Fabbro M, Rouays-Mabit H, Verollet C, Bauchet L. Metastatic Paraplegia and Functional Outcomes: Perspectives and Limitations for Rehabilitation Care. Part 2. *Archives of Physical Medicine and Rehabilitation.* 2011;92(1):134-145.
2. Hirabayashi H, Ebara S, Kinoshita T, et al. Clinical outcome and survival after palliative surgery for spinal metastases - Palliative surgery in spinal metastases. *Cancer.* 2003;97(2):476-484.
3. Eriks IE, Angenot EL, Lankhorst GJ. Epidural metastatic spinal cord compression: functional outcome and survival after inpatient rehabilitation. *Spinal Cord.* Apr 2004;42(4):235-239.
4. Fattal C, Fabbro M, Gelis A, Bauchet L. Metastatic Paraplegia and Vital Prognosis: Perspectives and Limitations for Rehabilitation Care. Part 1. *Archives of Physical Medicine and Rehabilitation.* 2011;92(1):125-133.
5. Outcomes following spinal cord injury: A Clinical Practice Guideline for Health-Care Professionals. In: Medicine CfSC, ed. Washington, D.C.: Paralyzed Veterans of America; 2005.

[*] SCI Model Systems For more information, refer to: http://www.spinalcord.uab.edu/show.asp?durki=104757&site=1226&return=21392

[††] CARF Accredited SCI Programs in California: For more information, refer to: http://www.carf.org/advancedProviderSearch.aspx

6. Metastatic spinal cord compression: diagnosis and management of patients at risk of or with metastatic spinal cord compression. In: Britain) NIfHaCEG, ed. London: National Institute for Health and Clinical Excellence (NICE); 2008.

7. Stubblefield MD, Bilsky MH. Barriers to rehabilitation of the neurosurgical spine cancer patient. *J Surg Oncol.* Apr 2007;95(5):419-426.

8. Helweg-Larsen S, Sorensen PS, Kreiner S. Prognostic factors in metastatic spinal cord compression: a prospective study using multivariate analysis of variables influencing survival and gait function in 153 patients. *Int J Radiat Oncol Biol Phys.* Mar 15 2000;46(5):1163-1169.

9. Abrahm JL, Banffy MB, Harris MB. Spinal cord compression in patients with advanced metastatic cancer: "all I care about is walking and living my life". *Jama.* Feb 27 2008;299(8):937-946.

10. Bagnall AM, Jones L, Richardson G, Duffy S, Riemsma R. Effectiveness and cost-effectiveness of acute hospital-based spinal cord injuries services: systematic review. *Health Technol Assess.* 2003;7(19):iii, 1-92.

11. Ruff RL, Adamson VW, Ruff SS, Wang X. Directed rehabilitation reduces pain and depression while increasing independence and satisfaction with life for patients with paraplegia due to epidural metastatic spinal cord compression. *J Rehabil Res Dev.* 2007;44(1):1-10.

Non-specific Low Back Pain

	Behaviors
**1**	For patients with new onset (<15 days) non-radicular LBP and mobility deficits, thoracic, lumbar and/or pelvic girdle mobilization and manipulation procedures should be provided when deemed appropriate by the treating therapist. If a patient meets these criteria but is not provided with mobilization or manipulation, the reason should be documented. (Strong)[1-4]
**2**	Patients with non-specific LBP should be assessed for lumbar instability based on the following criteria: (1) positive prone stability test, (2) positive (>6/9) Beighton scale[‡], (3) aberrant movement patters (instability catch, Gower sign), (4) production of pain with mobilization of hypermobile segment (especially L4-5, and L5-L1), and/or (5) presence of excessive lumbar mobility (excessive lumbar flexion/reversal of lumbar lordosis). [4-6]
**3**	Patients with lumbar instability who have 3 or more of the following: (1) age <40, (2) SLR >91°, (3) positive prone instability test, or (4) aberrant movement during lumbar spine active range of motion (Gower sign, instability catch, reversal of lumbo-pelvic rhythm), should be provided with lumbar stabilization exercises. If a patient meets these criteria but is not provided with lumbar stabilization exercises, the reason should be documented. (Strong) [4]
**4**	For patients with radiating LBP secondary to discogenic pain, repeated movement exercises should be tested for effectiveness of symptom centralization (distal to proximal movement of symptoms). If the exercises are effective they should be prescribed as part of a specific exercise program.[7] (Strong)
**5**	For patients with acute or non-radicular LBP, traction should NOT be provided unless a specific rationale is provided. (Traction may be indicated for patients with radicular symptoms who do not respond to repeated movements exercises.) [3,4,8,9](Medium)

[‡] The Beighton score is measured by adding 1 point for each of the following: 1) Placing flat hands on the floor with straight legs; 2) Left knee hyperextension; 3) Right knee hyperextension; 4) Left elbow hyperextension; 5) Right elbow hyperextension; 6) Left thumb touching the forearm ; 7) Right thumb touching the forearm; 8) Left little finger hyperextension past 90 degrees; 9) Right little finger hyperextension past 90 degrees

**6**	For patients with radiating LBP symptoms, neural mobility techniques should be provided during the course of care when deemed appropriate by the treating therapist. If a patient meets these criteria but is not provided with neural mobility techniques, the reason should be documented. [4](Strong)<br>***Reviewer Comments:*** The *cited evidence addresses patients with sub-acute and chronic presentation. Due to potential irritability of the nervous system, caution should be used for patients with acute presentation. Presence or absence of concurrent neurological signs could necessitate more intensive diagnostic workup such as MRI.*
**7**	For patients with chronic (>12 weeks) LBP, a progressive exercise program (neuromuscular control, strength, and endurance) should be provided. If a patient meets this criteria but is not provided with progressive exercises, the reason should be documented. [10](Strong)
**8**	For patients with acute non-specific LBP, advice to stay active (as opposed to bed rest) should be provided. The recommendation should be documented.[11] (Strong)
**9**	For patients with acute (<6 weeks duration) and sub-acute (6-12 weeks duration) LBP, back pain education (posture modification, biomechanics training, pain management) in group or individual learning modules should be offered. If a patient meets these criteria but is not offered back school education, the reason should be documented. [12](Strong)
**10**	For patients with chronic (>12 weeks duration) non-specific LBP, a multidisciplinary rehabilitation program (including exercise, psychological pain management, back school, PT/OT, psychology/psychiatry, and medical management) should be considered. Therapists should document discussion of the appropriateness of such an intensive program with patients with chronic non-specific LBP.[13](Strong)

1. Childs MJD, Fritz JM, Flynn TW, et al. A clinical prediction rule to identify patients with low back pain most likely to benefit from spinal manipulation: A validation study. *Annals of Internal Medicine.* 2004;141(12):920-928.
2. Flynn T, Fritz J, Whitman J, et al. A clinical prediction rule for classifying patients with low back pain who demonstrate short-term improvement with spinal manipulation. *Spine.* 2002;27(24):2835-2843.
3. Fritz JM, Lindsay W, Matheson JW, et al. Is there a subgroup of patients with low back pain likely to benefit from mechanical traction? Results of a randomized clinical trial and subgrouping analysis. *Spine.* Dec 2007;32(26):E793-E800.
4. Delitto A, George SZ, van Dillen L, Sowa G, Godges JJ. Low back pain clincial practice guidelines linked to the International Classification of Functioning, Disability, and Health. *J Orthop Sports Phys Ther.* in process.
5. Hicks GE, Fritz JM, Delitto A, McGill SM. Preliminary development of a clinical prediction rule for determining which patients with low back pain will respond to a stabilization exercise program. *Arch Phys Med Rehabil.* Vol 86. United States2005:1753-1762.

6. Hicks GE, Fritz JM, Delitto A, Mishock J. Interrater reliability of clinical examination measures for identification of lumbar segmental instability. *Arch Phys Med Rehabil.* Vol 84. United States2003:1858-1864.

7. Aina A, May S, Clare H. The centralization phenomenon of spinal symptoms--a systematic review. *Man Ther.* Aug 2004;9(3):134-143.

8. Fritz JM, Cleland JA, Childs JD. Subgrouping patients with low back pain: evolution of a classification approach to physical therapy. *J Orthop Sports Phys Ther.* Jun 2007;37(6):290-302.

9. Clarke J, van Tulder M, Blomberg S, de Vet H, van der Heijden G, Bronfort G. Traction for low back pain with or without sciatica: an updated systematic review within the framework of the Cochrane collaboration. *Spine (Phila Pa 1976).* Jun 15 2006;31(14):1591-1599.

10. Hayden J, van Tulder Maurits W, Malmivaara A, Koes Bart W. Exercise therapy for treatment of non-specific low back pain. *Cochrane Database Syst Rev.* 2005(3). http://www.mrw.interscience.wiley.com/cochrane/clsysrev/articles/CD000335/frame.html.

11. Dahm KT, Brurberg KG, Jamtvedt G, Hagen KB. Advice to rest in bed versus advice to stay active for acute low-back pain and sciatica. *Cochrane Database Syst Rev.* 2010(6):CD007612.

12. Engers Arno J, Jellema P, Wensing M, van der Windt Daniëlle AWM, Grol R, van Tulder Maurits W. Individual patient education for low back pain. *Cochrane Database Syst Rev.* 2008(1). http://www.mrw.interscience.wiley.com/cochrane/clsysrev/articles/CD004057/frame.html.

13. Henchoz Y, de Goumoens P, So AK, Paillex R. Functional multidisciplinary rehabilitation versus outpatient physiotherapy for non specific low back pain: randomized controlled trial. *Swiss Med Wkly.* 2010;140:w13133.

Post-Surgical Lumbar Spine

Post-Surgical Lumbar Spine	
**1**	For patients undergoing rehabilitation following spine fusion who present with severe pain and psychological impairments related to anxiety, depression, or chronic pain, patient education and counseling should be provided in addition to physical therapy for physical impairments.[1] (Medium: Intensive multidisciplinary rehabilitation including bio-psycho-social approach improves pain and function in severe chronic low back pain and provides as much benefit as spinal surgery. It can be used as an alternative to surgery, or it may be used to complement surgery.)<br><br>***Reviewer Comments:*** *Note that providing education on the psychological aspects of pain for those without psychological impairments may produce worse results then the match group that did not received that education.[A] Patient education and counseling on pain management is a skill that should administered by physical therapists, with or without the complementary use of psychotherapy intervention, for patients with LBP and concomitant mental impairments.[B]*<br>A. *George SZ, Fritz JM, Bialosky JE, Donald DA. The effect of a fear-avoidance-based physical therapy intervention for patients with acute low back pain: results of a randomized clinical trial. Spine. 2003;28:2551-2560. ("Patients with lower fear-avoidance beliefs appeared to have more disability from fear-avoidance–based physical therapy, when compared to those receiving standard care physical therapy.")*<br>B. *Godges JJ, Anger MA, Zimmerman G, Delitto A. Effects of education on return-to-work status for people with fear-avoidance beliefs and acute low back pain. Phys Ther. 2008;88:231-239*
**2**	For patients receiving post-operative care for laminectomy, neural mobilization is not indicated as an effective treatment to reduce pain or improve functional status.[2] (Low: In 1 RCT WITH 59 patients adding neural mobilization to a post-operative exercise program was not effective for pain and functional disability in the short or long term.)
**3**	For patients post-op lumbar spine surgery, high intensity trunk strength and endurance training should begin 4-6 weeks post-op.[2] (Strong: Rehab programs following lumbar disc surgery starting at 4 to 6 weeks post-surgery lead to a faster decrease In pain as well as high intensity exercise programs beginning at 4-6 weeks post op lead to faster decrease in pain than low intensity exercise.)

**4**	For post-operative spine patients, progressive lumbopelvic coordination training along with patient education and counseling in pain management that includes the following topics, 1) tissue healing, 2) physiological and psychological pain processes, 3) cognitive coping strategies, 4) positive reinforcement for progressive goal attainment, 5) action plan to manage hinders and flare-ups; should be initiated during the first post-operative week and continue for at least 3 more sessions spread out over 12 weeks."[3] (Strong: Psychomotor therapy, in addition to exercise therapy, produced significant improvements in functional disability, self-efficacy, outcome expectancy and fear of movement/re-injury compared to exercise alone. Psychomotor therapy produced 10 point larger decrease in Oswestry compared with exercise groups at all follow up intervals. They also had more employment and less long term sickness leave compared with the exercise therapy group.)
**5**	Post-operative spine patients should be encouraged to attend a low back surgery support group.[4] (Strong: A back-café group was the only group that had relatively low leg pain scores at both initial diagnosis and 2 year follow-up [better than video group or training group]. The social networking or inter-patient relationship created during the café group sessions might influence coping with pain.)

1. Guzman J, Esmail R, Karjalainen K, Malmivaara A, Irvin E, Bombardier C. Multidisciplinary bio-psycho-social rehabilitation for chronic low back pain. *Cochrane Database Syst Rev.* 2002(1):CD000963.

2. Ostelo RW, Costa LO, Maher CG, de Vet HC, van Tulder MW. Rehabilitation after lumbar disc surgery: an update Cochrane review. *Spine (Phila Pa 1976).* Aug 1 2009;34(17):1839-1848.

3. Abbott AD, Tyni-Lenne R, Hedlund R. Early rehabilitation targeting cognition, behavior, and motor function after lumbar fusion: a randomized controlled trial. *Spine (Phila Pa 1976).* Apr 15 2010;35(8):848-857.

4. Christensen FB, Laurberg I, Bunger CE. Importance of the back-cafe concept to rehabilitation after lumbar spinal fusion: a randomized clinical study with a 2-year follow-up. *Spine (Phila Pa 1976).* Dec 1 2003;28(23):2561-2569.
